# Is *Fucus* a suitable biomonitoring organism for polycyclic aromatic hydrocarbon contamination? A study from the Faroe Islands

**DOI:** 10.1007/s11356-024-32658-7

**Published:** 2024-03-08

**Authors:** Ida Huusmann Knøfler, Kirstine Evald Andersson, Richard Leonard Becker, Sigurd Christiansen, Nikoline J. Nielsen, Jan H. Christensen

**Affiliations:** 1 Department of Plant and Environmental Sciences, University of Copenhagen, Thorvaldsensvej 40, 1871 Frederiksberg C, Denmark; 2 Faculty of Science and Technology, University of the Faroe Islands, Vestara Bryggja 15, FO-100 Tórshavn, Faroe Islands

**Keywords:** Seaweed, PAHs, Marine pollution, Alkylated PAHs, GC–MS

## Abstract

To evaluate seaweed as a biomonitoring organism, *Fucus* was sampled in the Faroe Islands. Nineteen PAHs, including the EPA 16, and four groups of alkylated PAHs were quantified using GC–MS analysis of extracts obtained using a modified QuEchERS method with ultrasonication in acetonitrile, back-extraction into hexane, and Florisil® cleanup. Samples from the harbor of Tórshavn collected at high tide were the most polluted with PAH concentrations between 1.3 × 10^2^ and 1.7 × 10^2^ ng/g wet weight. All samples contained a factor 10 higher concentrations of alkylated PAHs compared to their parent compounds. These results suggest that *Fucus* might be suitable as a biomonitoring organism for PAH pollution. Differences between samples collected in close proximity and on different days were observed (same range of RSD 14–120% and 60–102%, respectively), suggesting that water exchange, tide levels, and direct exposure to surface diesel pollution have a strong influence on pollutant uptake in *Fucus*. The findings stress the need for further evaluation of the sampling strategy.

**Supplementary Information:**

The online version contains supplementary material available at 10.1007/s11356-024-32658-7.

## Introduction

Mid 1990’s activities related to the ocean have been the major driver of the economy in the Faroe Islands. Currently, fishing and its related industries account for more than 20% of the gross domestic product (The Government of the Faroe Islands 2019). Since 1995, aquaculture and shipping have contributed significantly to the economic growth. This economic expansion has led to increased traffic in the waters surrounding the Faroe Islands, increasing the pollution load, posing a threat to natural habitats and human health (Sorte et al. 2020).

One major group of pollutants related to the marine industries is polycyclic aromatic hydrocarbons (PAHs), which is a diverse group of hydrophobic, organic contaminants comprising at least two fused aromatic rings (Bergamasco et al. 2014; Lawal 2017). In the environment, they are often found with some degree of alkylation and are subjected to various transformations including biodegradation and photooxidation, which influences their physicochemical properties (Andersson and Achten 2015; Boll et al. 2015). PAHs are of environmental concern due to their specific toxicity towards aquatic life including fish, benthic organisms, and marine vertebrates (Honda and Suzuki 2020), not to mention their carcinogenic properties, posing a threat to human health (Ambade et al. 2021). In the Arctic marine environment, they originate from both natural sources including volcanic eruptions, underwater oil seeps and forest fires, and anthropogenic sources such as the use and combustion of coal- and oil-derived products (Balmer et al. 2019; Lawal 2017). The anthropogenic sources of PAHs vary from local sources to long-range transport with wind and ocean currents (AMAP 2004). The growing fishing and shipping industries are therefore also contributing to the increased levels of PAHs in the marine environment surrounding the Faroe Islands, due to the combustion of heavy fuels and diesel oil, and the use of coal tar for impregnation of wooden boats (Van Metre and Mahler 2010). The environmental fate of PAHs in the marine and terrestrial environments in the Arctic is of growing interest, and the understanding of the transport and degradation of PAHs in the Arctic ecosystems remain a scientific challenge (Balmer et al. 2019; Jörundsdóttir et al. 2014; Laender et al. 2011). Thus, it is of relevance to assess the occurrence and distribution of PAHs along the coasts of the Faroe Islands and to evaluate new and relevant biomonitoring organisms for exposure monitoring.

Biomonitoring organisms are used for quantitative determination of specific contaminants in the environment. They provide an integrated assessment of the presence of pollutants by only responding to the fraction of ecotoxicological relevance (Conti et al. 2008). An ideal biomonitoring organism for assessing local pollution needs to be easy to identify, sedentary, abundant, easy to collect, available to sample throughout the year, accumulator of the pollutant, and possible to analyse (Conti and Cecchetti 2003; Rainbow 1995). Blue mussels (*Mytilus edulis*) are considered ideal for in situ monitoring of lipophilic persistent organic pollutants in the marine environment, as they are sedentary filter feeders (Poulsen et al. 2021) but the accessibility can vary with the sampling location. The widespread occurrence of seaweed, its potential for reflecting passive uptake and its ability to tolerate pollutants makes it a potential alternative biomonitoring organism to blue mussels (Chalkley et al. 2019). Seaweed has been used for biomonitoring of trace metals via passive uptake routes, however the number of studies investigating the use of seaweed as a biomonitoring organism for PAHs are limited to a few recent studies on green, red, and brown macroalgae (Lourenço et al. 2019; Pavoni et al. 2003; Zokm et al. 2022). Furthermore, these studies have been carried out in the Mediterranean and sub-tropical climate, where the abundance of species, distribution, transport, and degradation of PAHs might differ from that of the Arctic.

*Fucus* is a genus of brown macroalgae which is highly abundant in the intertidal zones along the rocky coasts of the North Atlantic (Jueterbock et al. 2013). Members of the genera *Fucus* are canopy-forming macroalgae, which due to their large surface area provide liveable habitats for a large variety of marine organisms. Therefore, assessing the accumulation of PAHs in *Fucus* is not only relevant in relation to environmental monitoring, but also for environmental conservation. Knutzen and Sortland (1982), investigated the uptake of PAHs in *F. vesicolosus* along the coasts of Norway and found a total PAH concentration for up to 28 PAHs including methylated PAHs of 284–4665 µg/kg dry weight depending on the sampling location. Kirso and Irha (1998) assessed the role of red, green, and brown macroalgae in the distribution of PAHs in the Baltic Sea. Their results showed that 80 – 89% of the initial amount of benzo(a)pyrene added in an ex situ experiment was found in the biomass of *F. vesicolosus,* which suggests bioconcentration of PAHs. These findings indicate that *Fucus* as a native species might also serve as a suitable passive biomonitoring organism for PAHs in the Arctic (Conti and Cecchetti 2003; García-Seoane et al. 2018; Rainbow 1995).

The aims of this study were to investigate whether the brown macroalgae *Fucus* takes up and accumulate PAHs from the surrounding environment, and to assess if it can be used as monitoring organisms for PAH contamination in the Arctic and specifically in the Faroe Islands. This includes comparison of inter-day sampling of the same genera under similar conditions at the same location, and of the same genera sampled at different conditions and locations.

Seaweed belonging to the *Fucus* genus was sampled in Tórshavn, Runavík, Toftir, and Kirkjubøur, and analyzed within 48 h after sampling. The extraction method for PAHs from seaweed was developed based on a QuEChERS (Quick, Easy, Cheap, Effective, Rugged, Safe) method designed for extraction of PAHs from heavily pigmented fruits (Słowik-borowiec et al. 2022). The chemical analysis was carried out using gas chromatography-mass spectrometry (GC–MS) in selected ion monitoring (SIM) mode. In order to investigate whether the measured PAHs were adsorbed to the surface of the sampled seaweed or absorbed into the tissue, a thorough wash with acetonitrile (ACN) followed by analysis of the liquid and tissue was conducted on two of the samples in parallel with the regular extraction.

## Method

### Sampling

**Fig. 1:**
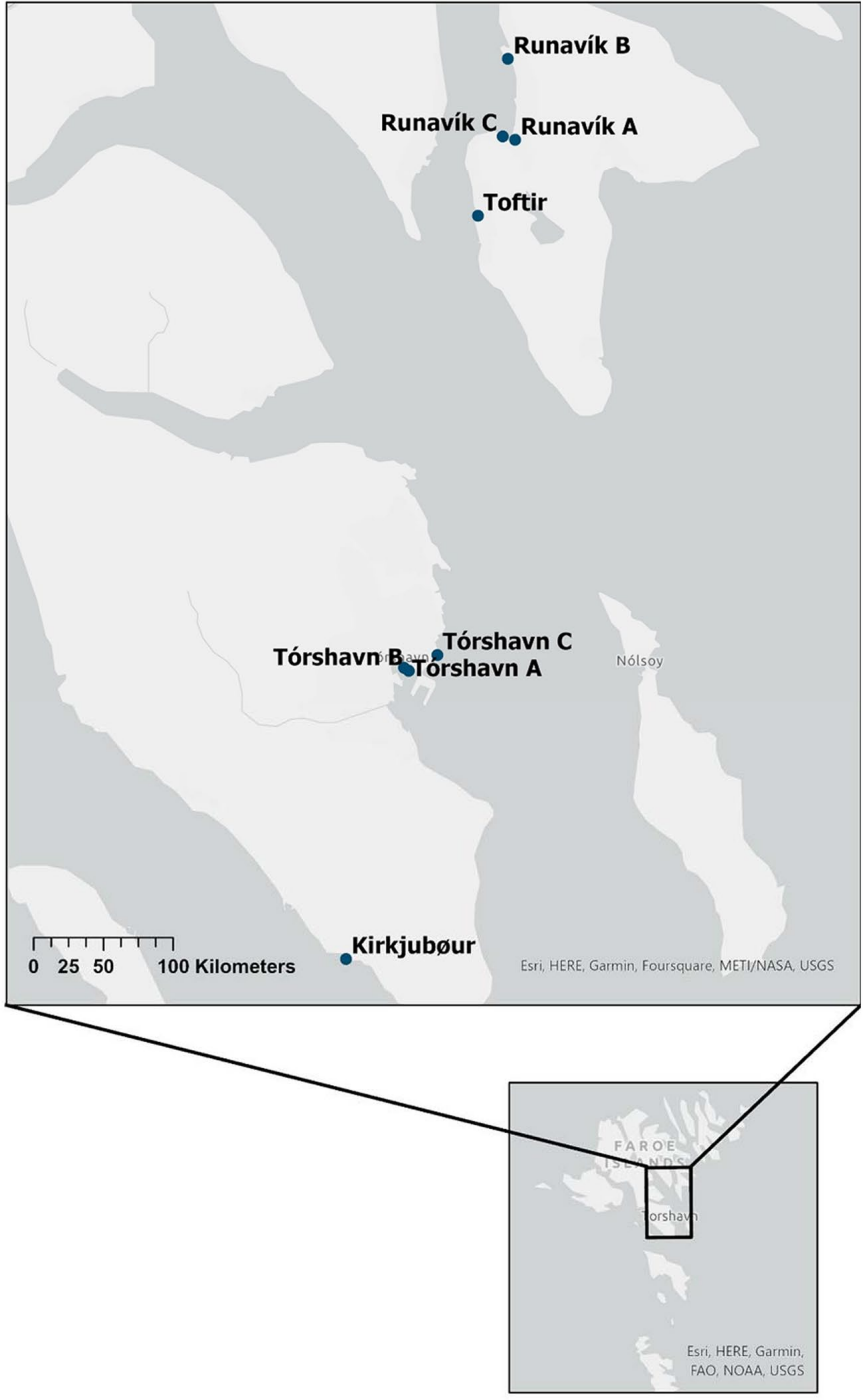
Sampling locations. Tórshavn A and B, and Runavík A and C were suspected hotspots for PAH pollution. Toftir and Runavík B were expected to be less polluted. Kirkjubøur and Tórshavn B were chosen as the reference sites. The samples THA-1–THA4 were sampled at Tórshavn A, THB-1 at Tórshavn B, and THC-1 at Tórshavn C. RA-1 + 2 were sampled at Runavík A, RB-1 at Runavík B, and RC-1 at Runavík C. TOA-1 was sampled at Toftir and KA1 + KB1 at the site in Kirkjubøur

Samples of seaweed were collected at six locations around Tórshavn (TH), Runavík (R), Toftir (TO), and Kirkjubøur (K) in the Faroe Islands (Fig. 1, table S1.1), between the 30 May 2022 and the 4 June 2022. Four of the locations (harbors in Runavík and Tórshavn) were suspected to be polluted with diesel oil and creosote originating from fishing and shipping activities. Kirkjubøur was suspected to be less polluted, due to its location on the west coast, where the water exchange is more rapid, and the density of marine traffic is lower. A sample area located northeast from the harbor of Tórshavn was also suspected to have a low concentration of PAHs and was therefore also sampled as a potential reference site. Toftir, a smaller harbor located south of Runavík, was suspected to be less polluted than Tórshavn and Runavík but have higher pollution than the reference sites. See supplementary information (figure S1.1) for detailed maps showing each sampling location. Each sample consisted of a composite sample from multiple individuals in the same area (maximum radius of 100 m) comprised of four to five increments each consisting of thalli from at least three individuals excluding stem and receptacles exceeding a length of 1 cm. The samples were consistently collected just below surface at the current water level.

#### Sample names

The two cities and two towns are represented with a 1–2 letter abbreviation Tórshavn (13,083 inhabitants (2017)) (TH), Runavík (3979 (2018)) (R), Toftir (793 (2017)) (TO), and Kirkjubøur (75 (2009)) (K). Different locations within the same city are sorted alphabetically (A, B, and C), and numbering refers to different samples collected at the same location. Further information about the samples can be found in Table 1.

**Table 1:** Sampling location, date, and modelled tide level

Samples	Location	Date	Time of sampling	Approximate tide level (m)	Relative tide level
THA1 + 2	Tórshavn A	30–05-2022	18:45	0.52	High
THA3 + 4	Tórshavn A	01–06-2022	11:30	0.20	Low
THB-1	Tórshavn B	04–06-2022	10:30	0.25	Low
THC-1	Tórshavn C	01–06-2022	16:15	0.30	Low
RA-1	Runavík A**	01–06-2022	17:30	0.40	High
RA-2	Runavík A**	04–06-2022	14:00	0.20	Low
RC-1	Runavík C**	04–06-2022	14:00	0.20	Low
RB-1	Runavík B**	04–06-2022	13:30	0.21	Low
TOA-1	Toftir**	01–06-2022	17:00	0.40	High
KA1 + KB1	Kirkjubøur	04–06-2022	15:00	0.41	Middle

Tide levels (m) given in relation to the lowest astronomic tide level (laveste astronomiske tidevand LAT)) according to Danish Meteorological Institute (DMI), 2021)

^**^Recorded tide levels for Tórshavn

### Standards, materials, and chemicals

Standard solutions for calibration containing 18 parent PAHs (EPA16 + benzo[e]pyrene and perylene) and 20 alkylated PAHs (table S5.4) were prepared in HPLC-grade iso-octane from Rathburn (Højbjerg, Denmark). The standard also includes dibenzothiophene and alkylated dibenzothiophene which are heterocyclic sulfur containing compounds. In the following sections, dibenzothiophene is included in the sum of the PAHs (∑PAH_19_). All samples were analyzed for the 19 parent PAHs. The ones present at concentrations above the detection limit (DL) are listed with concentrations in Table 2 and 3, and ∑PAH_19_ is the sum of the listed concentrations excluding those with concentrations below DL. An internal standard solution and a recovery solution containing nine and six deuterated PAHs, respectively (S5.4), each at a concentration of approximately 8 µg/mL, were also prepared in HPLC-grade iso-octane from Rathburn (Højbjerg, Denmark). The calibration, internal, and recovery standards used are listed in table S5.1, S5.2 and S5.3, respectively. Exact concentrations of the standard solutions are listed in supplementary information (table S5.5). Hexane (mixtures of isomers) and ACN (HiPerSolv) were both HPLC grade and purchased from VWR Chemicals (Søborg, Denmark). Primary secondary amine (Spera™ PSA) was acquired from Phenomenex (Værløse, Denmark). Florisil® (60–100 mesh) was acquired from Supelco (Søborg, Denmark). Sodium hydrogencitrate sesquihydrate (98.5 purity) (Na_2_C_6_H_6_O_7_) was purchased from Sigma-Aldrich (Søborg, Denmark), tri-sodium citrate dihydrate (99.0 purity) (Na_3_C_6_H_5_O_7_) was acquired from VWR (Søborg, Denmark), and magnesium sulphate (MgSO_4_) (98.0% purity) was purchased from ChemSolute (Roskilde, Denmark).

**Table 2:** PAH concentrations in nanogram per gram wet weight (ng/g ww)

Sample name	Σ PAH_19_	Naphthalene	Fluorene	Dibenzo-thiophene	Phenanthrene	Anthracene ****	Fluoranthene	Pyrene	Chrysene	Ant/ (Ant + Phe)	Flr/ (Flr + Pyr)
		Concentration (ng/g ww)									
THA-1	1.3 × 10^2^	0.41	15	2.0	62	-	3.1	40	1.8	**0**	**0.07**
THA-2	1.7 × 10^2^	0.48	23	2.6	94	-	4.4	47	1.9	**0**	**0.08**
THA-3**	2.5	< DL	< DL	< DL	0.69***	3.3 × 10^−2^	0.23	1.8	0.31	**0.04**	**0**
THA-4	57	0.38***	7.5	0.69	28	0.3	1.9	17	0.55	**0.01**	**0.10**
THB-1	0.31	< DL	< DL	< DL	< DL	-	9.8 × 10^−2^ ***	0.22***	< DL	**-**	**0**
THC-1*	0	< DL	< DL	< DL	< DL	-	< DL	< DL	< DL	**-**	**-**
RA-1	12	0.25***	< DL	0.40	6.1	-	0.54	3.3	1.0	**0**	**0.1**
RA-2	3.8	< DL	< DL	7.5 × 10^−2^ ***	1.1	1.1	0.14***	1.1	0.30	**0.5**	**-**
RC-1	0	< DL	< DL	< DL	< DL	-	< DL	< DL	< DL	**-**	**-**
RB-1	0	< DL	< DL	< DL	< DL	-	< DL	< DL	< DL	**-**	**-**
TOA-1	12	1.8	0.45***	0.34	5.7	-	0.40	2.0	0.53	**0**	**0.2**
KA-1*	0	< DL	< DL	< DL	< DL	-	< DL	< DL	< DL	**-**	**-**
KB-1*	0	< DL	< DL	< DL	< DL	-	< DL	< DL	< DL	**-**	**-**

^*^Reference site

^**^Sampled only from first sub-sample area at Tórshavn A

^***^Below LOQ but above DL

^****^DL was not calculated

**Table 3:** Concentrations of C1–C4 alkylated PAHs. C1–C4 alkylated naphthalene, C1–C3 alkylated phenanthrene, C1–C2 alkylated fluorene, and C1 alkylated pyrene were included in this study

Sample name	ΣPAH_Alk_	Alkyl. Naph (C1–C4)	Alkyl. Phen. (C1–C3)	Alkyl. Flourene (C1 + C2)	C1 Pyrene
	Concentration (ng/g ww)				
THA-1	1.5 × 10^3^	7.4 × 10^2^	4.0 × 10^2^	2.9 × 10^2^	27
THA-2	1.9 × 10^3^	9.8 × 10^2^	4.9 × 10^2^	3.6 × 10^2^	32
THA-3	69	28	27	10	3.4
THA-4	9.7 × 10^2^	5.8 × 10^2^	2.1 × 10^2^	1.7 × 10^2^	13
THC-1	17	5.6	7.1	3.0	1.2
THF-1	2.4	2.4	-	-	-
RA-1	1.4 × 10^2^	23	93	23	5.9
RA-2	55	8.8	37	7.2	2.3
RC-1	2.4	2.1	0.27	-	-
RB-1	4.6	2.8	1.8	-	-
TOA-1	78	25	38	12	2.7
KA-1	2.6	2.6	-	-	-
KB-1	2.2	2.2	-	-	-

### Method development

The method for sample preparation was based on a modified QuEChERS method for the extraction of PAHs in highly pigmented fruits and vegetables with a water content between 20 and 80% (Goodman 2011; Słowik-borowiec et al. 2022). During the method development, the extraction solvents ACN and *n*-hexane:acetone 4:1 (v/v) were tested, and ACN showed to be the more efficient solvent for the extraction of PAHs. Extraction by ultrasonication showed to be more efficient than dispersion with Ultra Turrax®, which was also tested. For the up-concentration and additional sample cleanup, a liquid–liquid back-extraction with hexane in ACN was preferred over up-concentration of *n*-hexane:acetone (4:1) v/v by evaporation, as it was less time consuming, and ACN was the better extraction solvent. Excess pigments were cleaned up by Florisil®, as it showed to be more efficient at removing pigments without removing PAHs compared to graphitized carbon black (GCB) and C18 fused silica. See supplementary information (S6) for a detailed description of the results from the method optimization.

### Sample preparation

To avoid contamination of PAHs after sampling, all samples were collected in Teflon bags and stored in a refrigerator at 5 °C within 2 h after sampling. Approximately 100 g of sample was homogenized using a small kitchen blender (Køkkenchef, 500 mL, 300 W, 10069036) after thorough cleaning with tap water to remove epiphytes, particles, and other organisms attached to the sample. Approximately 12.5 g of the homogenized sample was transferred to a 50-mL glass centrifuge tube, and 12.5 mL of ACN and 80 µL of internal standard solution were added. After mixing the solvent with the sample, the samples were placed in an ultrasonication bath for 30 min. After sonication, 1.8 g NaCl, 1.8 g Na_3_C_6_H_5_O_7_, and 0.9 g Na_2_C_6_H_6_O_7_ were added to the centrifuge tube, vortexed for 1 min, and afterwards centrifuged at 2000 rpm for 3 min. Approximately 10 mL of the upper organic phase was transferred to a new 50-mL centrifuge tube where 2 mL of hexane and 12.5 mL of tap water were added, vortexed for 1 min, and centrifuged at 2000 rpm for 3 min. The hexane phase of 1–1.5 mL was transferred to a 15-mL glass centrifuge tube containing 80 mg MgSO_4_ to dry any excess water, and 334 mg Florisil® was added.^1^ The centrifuge tube was vortexed and centrifuged at 2000 rpm for 2 min. Approximately 1 mL of the clean extract was transferred to a 2-mL amber GC vial with screw cap, and 80 µL recovery standard was added. One hundred microliters was transferred to a GC amber vial with a 300 µL insert for analysis, and the remaining sample was stored at − 20 °C.

### Surface wash with ACN

A study was performed to investigate whether PAHs in *Fucus* were absorbed into the tissue or adsorbed to the surface. A total of 25 g of *Fucus* (previously cleaned with tap water) was transferred to a 50-mL centrifuge vial and shaken vigorously for 30 s with 25 mL ACN. The liquid was transferred to another 50-mL glass centrifuge tube, and 160 µL internal standard solution was added. This procedure was then repeated to a total of two extractions that were analyzed separately. Afterwards, 25 mL of tap water and 4 mL of hexane were added to the two ACN samples, and a liquid–liquid extraction was performed. Finally, 160 µL of recovery standard solution was added, vial-mixed, and an aliquot was transferred to an amber GC vial with a 300 µL insert for analysis. The ACN washed samples were left for drying in the fume hood and thereafter treated as the other samples for analysis. The rest of the sampled *Fucus* were homogenized and analyzed as well.

### Chemical analysis

All samples were analyzed using an Agilent Technologies 7890A GC system coupled to an Agilent Technologies 5975 inert XL EI/CI Mass Selective Detector*.* The samples were injected in splitless mode with an injection volume of 1 µL. The separation of the analytes was performed on a 30-m Zebron ZB5 capillary column (Phenomenex, 0.25-mm inner diameter × 0.25-µm film thickness). Helium was used as the carrier gas at a constant flow of 1.1 mL/min resulting in an average velocity of 38.2 cm/s. The initial temperature was 50 °C, which was held for 1 min and hereafter increased by 25 °C/min until 100 °C, followed by an increase to 315 °C with 12 °C/min. The temperature was kept at 315 °C for 7 min, which resulted in a total analysis time of 28 min. Electron ionization (EI) was performed using an electron energy of 70 eV. The temperature of the EI source was 230 °C and the quadrupole 150 °C. The mass spectrometer was operated in SIM mode for 21 selected mass-to-charge (*m/z*) ratios, ranging from 83 to 288 m/z. The data acquisition was divided into 12 SIM groups with a dwell time of 20 ms of each m/z (table S7.1).

### Quality control/assurance and detection limits

In every batch, at least one method blank (containing 12.5 mL tap water) was treated the same way as the sample, from homogenization to addition of recovery standard, and GC–MS analysis.

To evaluate inter-day precision, a quality control (QC) sample was included in each batch and handled the same way as the regular samples (QC1). The QC1 sample consisted of 12.5 g homogenized *Saccharina latissima* from multiple individuals and was spiked with 240 µL 1:10 diluted stock solution, containing the 19 PAHs. In this case *S. latissima* tissue was used as it was deemed to have a comparable matrix to *Fucus* and due to its large thalli which made it more feasible to get a large enough sample to have several QC1 samples for analysis. DL and limit of quantification (LOQ) were assessed by preparing and analysing a batch of five QC2 samples consisting of a homogenized blend of *Fucus* sampled on the 4 June 2022 which contained a 10 times dilution of a PAH_19_ standard solution (table S5.5). The QC2 batch was analyzed 6 June 2022. The five samples of *Fucus* were also used for intra-day precision. The results are listed in supplementary information S3.

### Data treatment and quantification

MassHunter Workstation (Agilent Technologies, ver. B.07.00, 2014, USA) was used for quantification of PAHs. Four-level internal standard curves were used for quantification of each of the PAHs. Linear calibration curves with zero intercept were used as most samples contained target PAHs below the lowest standards. Concentrations were corrected for recovery determined from the surrogate standards added to the sample before extraction according to Poulsen et al. (2021). Alkylated PAHs were quantified based on the patterns exhibited by isomers of one alkylation level as described in the draft of the European Committee for Standardization guideline prEN 15522–2 (Dahlmann and Kienhuis 2016). If the patterns matched, all peaks were integrated and the signal was quantified with the help of the corresponding standard. When a major change in pattern, for example, a drastic increase in peak area for a single peak, was observed, this peak was excluded and the integration window terminated before or started after that peak. All graphs and plots were made using OriginPro 2020 (OriginLab Corporation, 2022). Maps illustrating the sampling locations were created using the ArcGIS Pro 2.5.1©2020 ESRI Inc.

### Diagnostic ratios

As a strategy for identifying the source of PAH pollution in the samples, two different diagnostic ratios were calculated. An Ant / (Ant + Phe) ratio < 0.1 classifies the source as petrogenic whereas a ratio > 0.1 classifies the source as pyrogenic. For a Flr / (Flr + Pyr) (Flr = fluoranthene) ratio < 0.4, the source is classified as petrogenic. For a ratio 0.4–0.5, the source is classified as fossil fuel combustion and > 0.5 ratio classifies the source as grass, wood, and coal combustion. The diagnostic ratios were calculated based on suggested ratios from Tobiszewski and Namieśnik (2012).

## Results

### PAH concentrations and diagnostic ratios

Table 2 shows ∑PAH_19_ concentrations in *Fucus* collected at the 13 sampling sites and two diagnostic ratios. The highest ∑PAH_19_ concentrations and of the most abundant individual PAHs were as follows: Tórshavn > Runavík > Toftir > reference sites. The highest ∑PAH_19_ concentrations were quantified in samples from Tórshavn (1.3 × 10^2^ ng/g ww), which is consistent with the suspected pollution level. Concentrations of PAHs at the reference sites were < DL. This was also observed for RC-1 and RB-1. For THB-1, none of the measured PAH concentrations exceeded LOQ.

In Tórshavn, the highest concentrations were observed for phenanthrene and pyrene, but relatively high concentrations (> 10% of ∑PAH_19_) of fluorene were observed in samples from THA-1, THA-2, and THA-4 (Table 2). The high concentrations of phenanthrene compared to anthracene and pyrene compared to fluoranthene demonstrate that the source of PAHs in the Tórshavn samples is of petrogenic origin (De La Torre-Roche et al. 2009; Pies et al. 2008), which is consistent with the observations of diesel spills on the water surface at the site.

In Runavík and Toftir, the same trend was seen where phenanthrene and pyrene were the highest ∑PAH_19_ concentrations. A high naphthalene concentration of 1.8 ng/g ww was found at TOA-1 compared to 0.25–0.48 ng/g ww at the 12 other sites. Likewise, high relative anthracene concentrations were observed at RA-2, which indicates a mixed source or another source than diesel oil. The fluoranthene-to-pyrene ratio is however lower than the limit of 0.4 which indicates that the PAHs are of mainly pyrolytic origin (De La Torre-Roche et al. 2009). The other samples from Runavík and Toftir are suspected to originate from petrogenic sources.

High variability was observed within sites of close proximity and samples from the same area. THA-1 and THA-2 showed the highest concentration of all ∑PAH_19_ (except for anthracene) but THA-3 and THA4 had 2–69 times lower concentrations although they were collected in the same area but two days later where the tide level differed 0.3 m (Table 1 and 2). The same was the case for RA-1 and RA-2 that were sampled 3 days apart also at high and low tide.

The mean RSD of ∑PAH_19_ between samples collected in close proximity at the same day (14% and 120% for THA1 vs THA2 and THA3 vs THA4) was in the same range as the RSD of samples from the same locations collected at different time points (between 60 and 102% RSD for THA1 vs. THA3, THA1 vs. THA4, THA2 vs. THA3, THA2 vs. THA4, and RA1 vs. RA2). For more details, see Table S8.1 in supporting information S8.

**Fig. 2:**
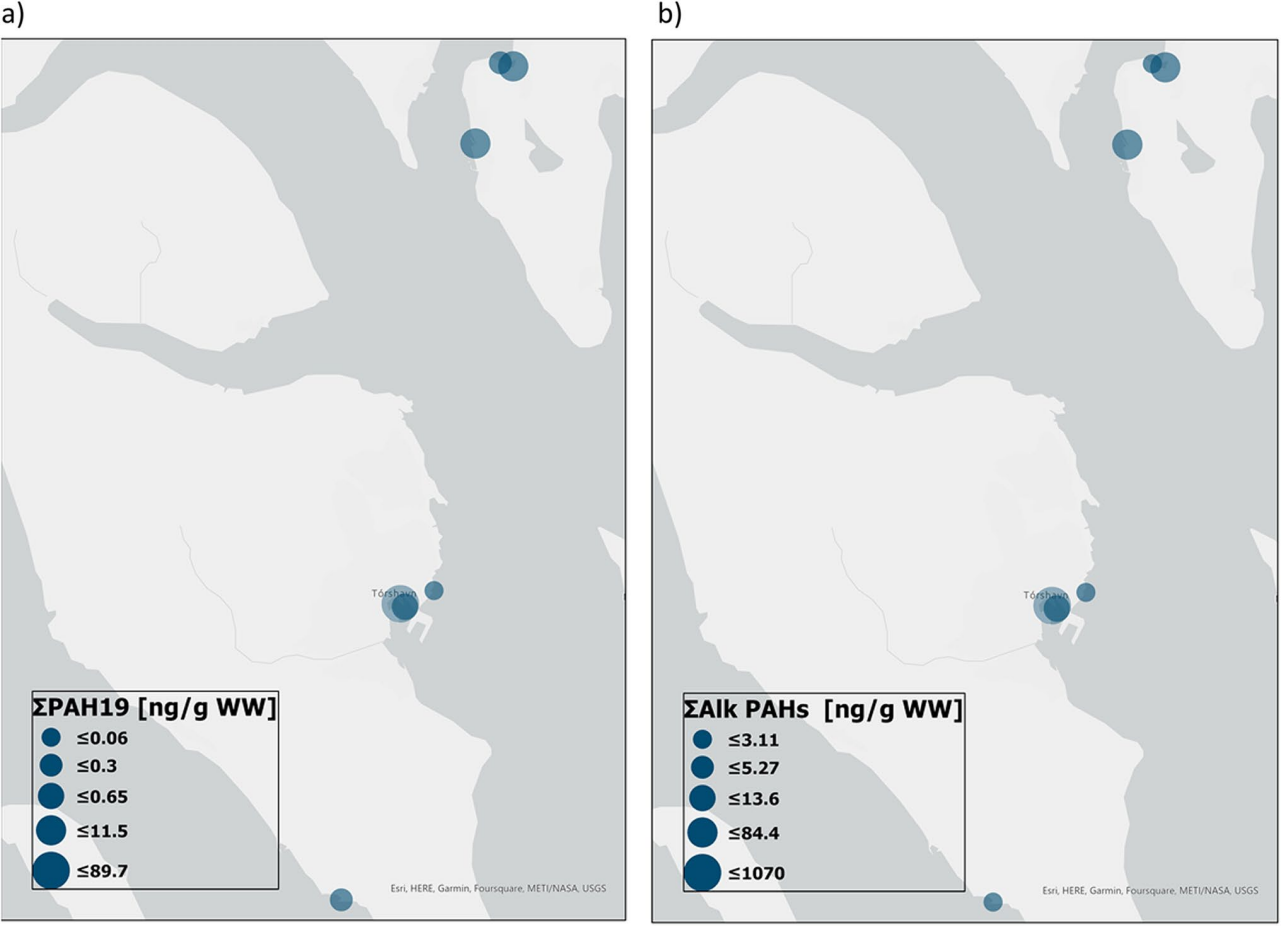
**a**, **b** Graphical representation of **a** ΣPAH_19_ and **b** ΣPAH_Alk_ (ng/g ww) at the sampling sites. The distribution of parent PAHs and the alkylated homologs follow the same trend. ΣPAH_19_ and ΣPAH_Alk_ include dibenzothiophene

Table 3 shows concentrations of alkylated PAHs at all sampling sites. For the alkylated PAHs, the same trend follows as for the parent compounds where the highest concentrations of ∑PAH_alk_ were as follows: Tórshavn > Runavík > Toftir > reference sites. The ∑PAH_alk_ is more than a factor 10 greater than ∑PAH_19_ for all samples except for TOA-1 where the ∑PAH_alk_ to ∑PAH_19_ ratio is 6.7. The sample with the lowest ∑PAH_alk_ concentrations was the same sample with the lowest ∑PAH_19_, RC-1, and RB-1 (2.4 and 4.6 ng/g ww, respectively). In contrast, THB-1 with a low ∑PAH_19_ of 0.31 ng/g ww showed 51 times higher ∑PAH_alk_ (17 ng/g ww) compared to the reference sites (2.4, 2.6, and 2.2 ng/g ww). For the Tórshavn samples, the highest concentration of alkylated PAHs was observed for alkylated naphthalenes even though the parent PAH is observed in low concentrations. The concentration of alkylated PAHs follows the same trend in concentration as observed for the parent PAHs: THA-2 > THA-1 > THA-4 > THA-3 > THB-1 (Table 3). The same is the case across sites as illustrated in Fig. 2.

For Runavík and Toftir, the most abundant alkylated PAHs were C2 phenanthrenes (data not shown). The distribution between alkylated PAHs at the sites indicates the degree of mainly evaporative weathering where less low-molecular-weight alkylated PAHs are observed (Wang and Fingas 1995). This effect is especially relevant for naphthalene with a steep increase from C1 to C4 naphthalenes.

**Fig. 3:**
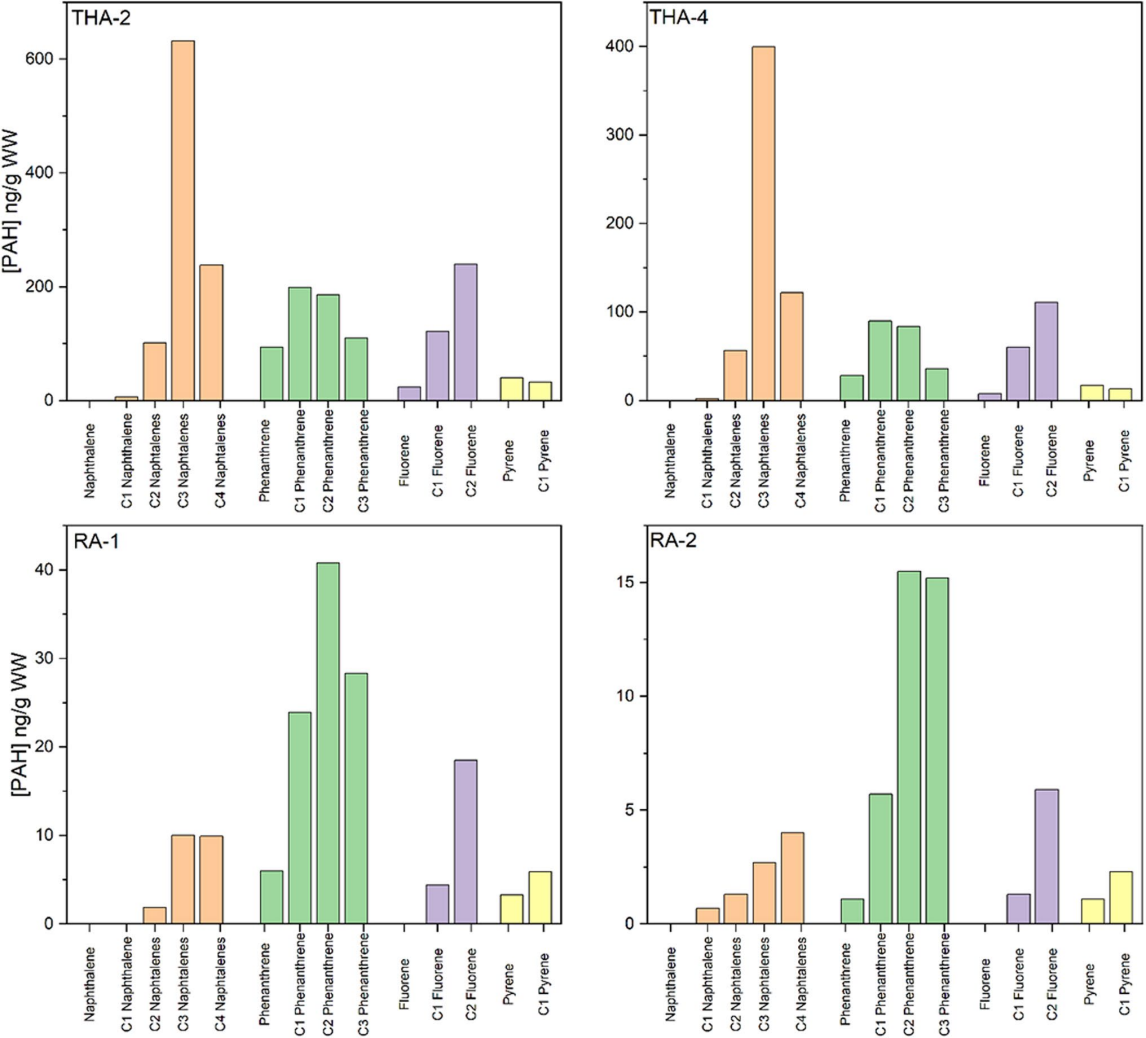
Comparison of the concentrations of parent and alkylated PAHs in high concentration samples from Tórshavn (THA-2 and THA-4) and in low concentration samples from Runavík (RA-1 and RA-2)

Figure 3 compares the relative composition of C1–C4 alkylated PAHs between two samples with high ∑PAH_alk_ from Tórshavn and two samples from Runavík with low ∑PAH_alk_. The alkylated PAH profiles were similar within each of the sites although they were sampled at different days, which indicate same source and similar degree of evaporative weathering. C3-alkylated naphthalenes were the most abundant alkylated PAH group in the samples from Tórshavn. The Runavík samples have higher relative concentration of alkylated phenanthrenes compared to naphthalenes indicating a heavier petroleum product or a more weathered diesel oil sample. The low concentration of alkylated PAHs at Runavík should be considered when evaluating the PAH profiles as a small absolute error will produce a large relative difference.

### ACN-washed Fucus

The PAH concentrations (∑PAH_19_ and ∑PAH_alk_) in the washed tissue and in the washing liquid are listed in Table 4. ∑PAH_19_ and ∑PAH_alk_ in the washing liquid were lower compared to the washed tissue. The diagnostic ratios of the washed tissue are comparable with the tissue that had not been washed (data not shown). The concentration of all PAH_19_ in the washing liquid for THA-3 was < DL, and only phenanthrene and pyrene were > DL (0.47 ng/g ww and 0.78 ng/g ww, respectively) in THA-4 where a decrease in concentrations was observed from the first to the second wash (analyzed ACN). ∑PAH_alk_ > ∑PAH_19_ in both the washing liquid and in the tissue but same trend was observed where ∑PAH_alk_ in the tissue > ∑PAH_alk_ wash 2 > ∑PAH_alk_ wash 1.

**Table 4:** Concentrations of alkylated PAHs and the PAH_19_ in *Fucus* tissue and ACN

Sample name	THA-3 Washed tissue	THA-3 Wash 1	THA-3 Wash 2	Sum	THA-4 Washed tissue	THA-4 Wash 1	THA-4 Wash 2	Sum
	Concentration (ng/g ww)							
Σ PAH_19_	**2.0**	**–**	**–**	**2.0**	**36**	**1.2**	**–**	**37**
Naphthalene	< DL	< DL	< DL	**–**	< DL	< DL	< DL	**–**
Fluorene	< DL	< DL	< DL	**–**	3.4	< DL	< DL	**3.4**
Dibenzothiophene	< DL	< DL	< DL	**–**	0.48	< DL	< DL	**0.48**
Phenanthrene	0.86	< DL	< DL	**0.86**	15	0.47*	< DL	**16**
Fluoranthene	0.12*	< DL	< DL	**0.12**	1.5	< DL	< DL	**1.5**
Pyrene	0.91	< DL	< DL	**0.91**	15	0.78	< DL	**15**
Chrysene	0.12	< DL	< DL	**0.12**	0.45	< DL	< DL	**0.45**
ΣPAH_Alk_	**56**	**4.5**	**0.82**	**62**	**8.6 × 10**^**2**^	**32**	**9.6**	**9.0 × 10**^**2**^
Alkyl. Naph. (C1–C4)	16	0.58	1.4** × **10^–2^	**27**	3.5** × **10^2^	6.4	2.8	**3.6 × 10**^**2**^
Alkyl. Phen. (C1–C3)	18	3.4	0.74	**22**	2.8** × **10^2^	17	4.3	**3.0 × 10**^**2**^
Alkyl. Flourene (C1 + C2)	19	–	–	**19**	2.1** × **10^2^	6.3	1.9	**2.1 × 10**^**2**^
C1 Pyrene	2.6	0.58	–	**3.2**	21	1.8	0.56	**23**

The washing liquid had a green color after 30 s of shaking, which indicates that pigments from inside the *Fucus* were also extracted. This suggests that the experiment was thorough in washing the outside of the tissue. An important conclusion from this is that the majority of the observed PAHs seem to be absorbed deeper inside the tissue of *Fucus* and not simply adsorbed to the surface. This emphasizes the relevance of the PAH results from this study in terms of evaluation of *Fucus* as a biomonitoring organism.

For the washed tissue, the ∑PAH_19_ concentration was lower in both samples compared to the tissue that has not been washed with can, and no clear trend is seen where specific PAHs are decreasing in concentration after the wash, which indicates that the wash with ACN was not selectively washing away certain PAHs. All the PAHs found in Table 2 and 3 for THA-3 and THA-4 could not be traced in the combined washed tissue and washing liquid. A higher concentration of alkylated fluorene (C1 and C2) was observed in the washed tissue compared to the tissue not washed with ACN in THA-3 (19 vs. 10 ng/g ww). For THA-4, all PAH_alk_ except for naphthalenes were higher in the washed tissue compared to the tissue washed only with tap water.

## Discussion

### PAH concentrations in Fucus

The results indicate that *Fucus* takes up PAHs and that it can be used as a biomonitoring organism for parent and alkylated PAHs in the marine environment. Other studies examining PAH concentrations in *Fucus* or comparable brown algae growing in the intertidal zone found between 88 and 2010 ng/g dw total PAHs in *S. furactum* on the coast of Brazil (Lourenço et al. 2019), 349–2109 ng/g dw napthalene and methylnaphthalenes as well as 284–4665 ng/g dw of total PAHs in *F. vesiculosus* samples from the coast of Norway (Knutzen and Sortland 1982) and 2–5 ng/g dw total PAHs where phenanthrene was the most abundant (29%) in *F. virsoides* from the lagoon of Venice (Pavoni et al. 2003). When comparing PAH content in living tissue, the most common way is to compare measurement per dry weight (dw). Water content was, however, not measured for all samples in this study. The conversion from g ww to dw is therefore estimated based on the water content from selected individuals of *Fucus* (mean water content 83%, SD ± 1.8) (table S4.1) in order to compare them with other studies. The phenanthrene found in this study converted with the water content estimate to 465 ng/g dw and is much higher than the most comparable study from Pavoni, but in the range given by the other studies.

The take-up of PAHs is underestimated if only parent PAHs are considered. The inclusions of alkylated PAHs increases the total PAH concentration tenfold for most samples. Alkylated PAHs are not nearly as well researched as their corresponding parent PAHs, and our results demonstrate that the inclusion of alkylated PAHs is important to avoid underestimation of PAH exposure to the marine environment especially when the main sources are of petrogenic origin such as diesel oil spills (Barron and Holder 2003; Du et al. 2022). Furthermore, some studies suggest that alkylated PAHs might pose a larger threat to the environment and human health than their parent compounds (Kang et al. 2016; Lam et al. 2018; Sun et al. 2014). Because of the low interference of matrix components and high concentration of mixtures of alkylated PAHs observed in this study, an unexpected benefit from examining *Fucus* might also be the possibility of comparing the alkylated PAH concentrations with the concentration of the corresponding parent PAHs.

A clear difference of PAH concentration in *Fucus* is seen between the suspected high concentration areas compared to the suspected low concentration reference sites. For *Fucus* sampled at location 4 in Tórshavn and at location 2 in Runavík, the concentrations are comparable to the low concentrations found at the reference sites. This indicates that the pollution might be very local. The difference in PAH concentration in *Fucus* that was sampled at close proximity at different days is suspected to be explained by at least one of the three factors: bioconcentration, water exchange, and exposure to the source of PAHs.

The comparison between samples within this study is constrained by the limited availability of data explained by the low number of samples. Therefore, the conclusions drawn from the results of this study are related to a high degree of uncertainty. The interpretation of the results presented in the following represents trends and descriptive insights in the differences between samples rather than statistically validated conclusions.

#### Bioconcentration

The low concentrations or absence of PAHs in the extraction solvent after both the first and second surface wash with ACN (wash 1 and wash 2) and the high concentrations of PAHs in the washed tissue indicate that both the parent and alkylated PAHs are absorbed by *Fucus.* According to Kirso and Irha (1998), *Fucus vesiculus* can bioconcentrate benzo(a)pyrene with a very high uptake and only low degradation 10 days after the exposure in ex situ experiments. Assuming different species in the *Fucus* genus behave similarly, the results indicate that *Fucus* is able to accumulate PAHs with limited degradation.

#### Water exchange and exposure

The exposure and water exchange seem to explain the variations in the PAH concentrations across days and between samples of close proximity. This is supported by results obtained by Garcia and Martins (2021) who examined the PAH concentration in sediment at sheltered and non-sheltered areas. They concluded that the PAH concentration is heavily affected by marine hydrodynamics, energy, and/or dilution processes and that the observed concentration was higher in areas with low water exchange.

This could explain the differences in PAH concentrations between two geographically close sampling locations (RA vs. RB and THA vs. THB), but does not, however, explain the inter-day concentration difference observed in *Fucus* at Tórshavn 1 and Runavík 1 (THA-1 and THA-2 > THA-3 and THA-4 and RA-1 > RA-2), which are both in sheltered harbors where water exchange is low and the concentration therefore not suspected to differ much from day to day.

Another factor influencing the exposure of *Fucus* to PAHs is the tide levels. The results show that samples from the same locations collected at different days with different tide levels (different time of the day) contain notably different levels of PAHs. THA-1 and THA-2, which were collected at high tide, show ∑PAH_19_ of 1.3 × 10^2^ and 1.7 × 10^2^ ng/g ww whereas THA-4, which was collected at low tide, contains ∑PAH_19_ of 57 ng/g ww (Table 2). Since the pattern of the alkylated PAHs is similar between THA-2 and THA-4 (Table 3), a similar source can be assumed. This is also the case for RA-1 (∑PAH_19_ = 12 ng/g ww) and RA-2 (∑PAH_19_ = 3.8 ng/g ww) that were collected at high and low tides, respectively.

Benes and Bracken (2016) demonstrated that *F. vesiculosus* growing in the upper intertidal zone has a significantly higher nutrient uptake rate compared to *F. vesiculosus* growing in the lower intertidal zone. The reason for this difference is that nutrient uptake only happens when the macroalga is submerged under water. Thus, individuals growing in the upper intertidal zone that are submerged for a shorter period have adjusted the uptake rate, so the total nutrient uptake is comparable to the individual growing in the lower intertidal zone. This increase in activity could also lead to increase in uptake of PAHs by *Fucus* growing in the upper intertidal zone, as these individuals are exposed to air during low tide, and in direct contact with the oil pollution on the surface of the water during high tide.

Thus, the results indicate that an important factor for accumulation of PAHs is the time of direct exposure to the PAHs such as a diesel oil slick on the water surface, which is probably why the *Fucus* sampled at high tide contains the highest ∑PAH_19_ and ∑PAH_alkyl_. The differences in PAH concentrations might thus be due to sampling at different tidal currents. All *Fucus* samples were collected from the water surface, independently of the time of day, and therefore, different individuals were picked, depending on the current water level in the harbor. This suggests that individuals growing at different tidal heights will not be accumulating the same amount of PAHs, and tidal height or growing height needs to be taken into account in order for inter-day samples to be comparable.

All samples have low naphthalene concentrations, which is more likely to dissolve in water than the larger PAHs, and more likely to evaporate from the surface (Baumard et al. 1999; Maxin and Kögel‐Knabner 1995; Whitehouse 1984). An additional experiment was conducted where the two kelp species, *S. latissima* and *Alaria esculenta*, growing further down the water column, from Tórshavn and Runavík, were analyzed for PAHs. For these samples, the PAH concentrations were below the DL except for phenanthrene, which was the most abundant parent PAH in that area and naphthalene, which is the most water soluble. Phenanthrene and naphthalene concentrations were just above DL in two *S. latissima* and one *A. esculenta* sample from Tórshavn (data not shown).

These results indicate that the seaweed must be in direct contact with the source of PAHs, in this case oil slicks on the water surface, in order to take them up. Therefore, individuals growing in the intertidal zone are more likely to reflect surrounding surface pollution. This could affect how well *Fucus* reflects the surrounding pollution and therefore how well it is suited as a biomonitoring organism. However, our results indicate that the concentrations of PAHs in *Fucus* reflect the source of PAHs as well as the suspected level of pollution on the sampling site. To support this, further research is needed to understand the interactions between *Fucus* and marine PAH pollution. An important aspect is investigation of how well the concentrations of PAHs in *Fucus* reflect the concentrations and composition of PAHs found in water. This investigation would also add to the understanding of *Fucus* as an accumulative passive bioindicator (Polechońska and Klink 2023). Other important investigations include elucidation of the effects of species, PAH sources, sampling depths, and tidal levels on the uptake of PAHs in macroalgae such as *Fucus*. It is too early to make a definite conclusion on the suitability of *Fucus* as biomonitoring organism. However, we hypothesize that the suspected up-concentration of PAHs in *Fucus* reflects the condition of the surrounding environment. The sampled species is the one factor where we can make the most direct assumptions about the effects of the measured PAH concentrations. Few studies focus on the direct effects of PAHs on kelp or *Fucus* in particular. One laboratory setup where *Fucus vesiculosus* was exposed to a mixture of diesel and seawater found only a short-term increase on the level of lipid peroxidation and no change to metabolic activity or catalase concentration (Ryzhik et al. 2019). Due to the short length of the study (6 days), no conclusion could be drawn about the overall health of the algae. Interestingly, the study found a 350% increase in hydrocarbon-oxidizing bacteria on the thalli of the algae when exposed to the diesel-water mixture. Another intertidal kelp species (*Lessonia spicata*) was found to be sensitive to PAHs especially during the sporophyte formation, with the EC_50_ for the inhibition of the formation of sporophytes being 0.04 µg/L PAH_EPA16_ though this species might be particularly susceptible to reactive oxygen species which are created in photosynthetic organisms when exposed to PAHs (Espinoza-González et al. 2021). As a primary producer and a foundation species, *Fucus* is a food source and an important habitat for many invertebrate species like *Idotea balthica* or *Tonina fluviatilis*, especially in the form of shed detritus. PAHs attached to the detritus could further contribute the PAH load on the sediment. These invertebrates were found to either directly graze on *Fucus* or eat its detritus (Kahma et al. 2023). Alkylated PAHs were found to also have similar toxic effects as parent PAHs to shrimp, microalgae, and a bacterial system although there might be further unidentified toxic mechanisms (Cong et al. 2021; Kang et al. 2016). This class of substances should be paid attention to as they were found in higher concentrations than their non-alkylated counterparts in this study and are the predominant form of PAHs in crude oil. Overall, it can be safely assumed that the *Fucus* and species directly connected to it in the ecosystem will be negatively affected by the exposure to PAHs. The magnitude of these effects is difficult to gauge due to the complexity of the exposure scenario.

## Conclusion

*Fucus* take up PAHs at measurable concentrations at sites with high PAH pollution levels such as the inner harbors of Tórshavn and Runavík in the Faroe Islands. These results suggest that *Fucus* is a suitable biomonitoring organism for PAH pollution. Our results however demonstrate that position in the inter-tidal zone and PAH sources are key factors that affect uptake of PAHs in *Fucus* and that it reflects only very local pollution levels. More research is needed to determine effects of the different factors on the uptake of PAHs. The differences in PAH concentrations in samples taken in close proximity could reflect very local surface water contamination. This hypothesized ability is an advantage; however, the assessment requires knowledge about the underlying hydrodynamics, water exchange, and dilution processes. Overall, the use of *Fucus* as a biomonitoring organism for PAHs and their alkylated homologues seems promising, but further criteria for the sampling strategy need to be assessed.

### Supplementary Information

Below is the link to the electronic supplementary material.

Supplementary file1 (DOCX 1181 KB)

## Data Availability

Further data supporting the findings of this study are available in Supplementary Information. Raw data are available from the corresponding author upon reasonable request.

## Footnotes

1 PSA was used in TFA-1, TFA-2, TAA, TLA, TLB, TLC, TLD, and TLE to further cleanup but was afterwards left out because of suspected pollution of pyrene and phenanthrene.
