# Supplementary material for: Is Fucus a suitable biomonitoring organism for polycyclic aromatic hydrocarbon contamination? A study from the Faroe Islands
Source: Environ Sci Pollut Res Int. 2024 Mar 8;31(18):26699–712. doi: 10.1007/s11356-024-32658-7 (PMC11052821; doi:10.1007/s11356-024-32658-7)
Supplement: Supplementary file 1 — Supplementary file1 (DOCX 1181 KB) [file 11356_2024_32658_MOESM1_ESM.docx]

# **Supporting Information (SI) - Is *Fucus* a Suitable Biomonitoring Organism for Polycyclic Aromatic Hydrocarbon Contamination? A study from the Faroe Islands**

**Authors:**

Ida Huusmann Knøfler*a

Kirstine Evald Andersson*a

Richard Leonard Becker*a

Sigurd Christiansen b

Nikoline Nielsen a

Jan H Christensen a

*Contributed equally to this work

a Department of Plant and Environmental Sciences, University of Copenhagen, Thorvaldsensvej 40, 1871 Frederiksberg C, Denmark

b Faculty of Science and Technology, University of the Faroe Islands, Vestara Bryggja 15,
 FO-100 Tórshavn, Faroe Islands

Contact information:

Jan H. Christensen

jch@plen.ku.dk

Thorvaldsensvej 40,

1871 Frederiksberg, DK

# S1.Sampling locations

| 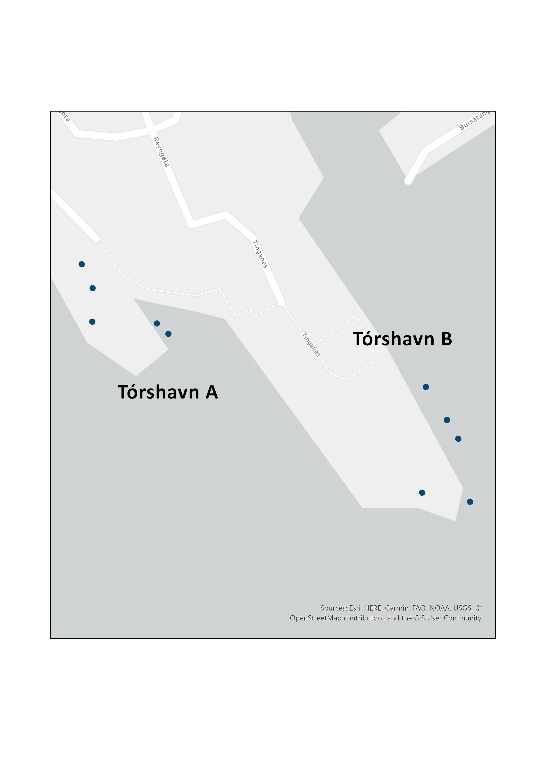 | 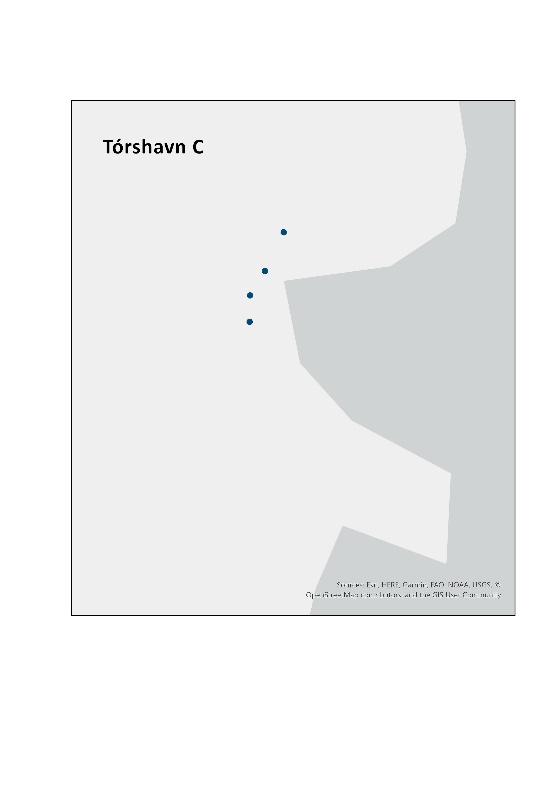 |
| --- | --- |
| 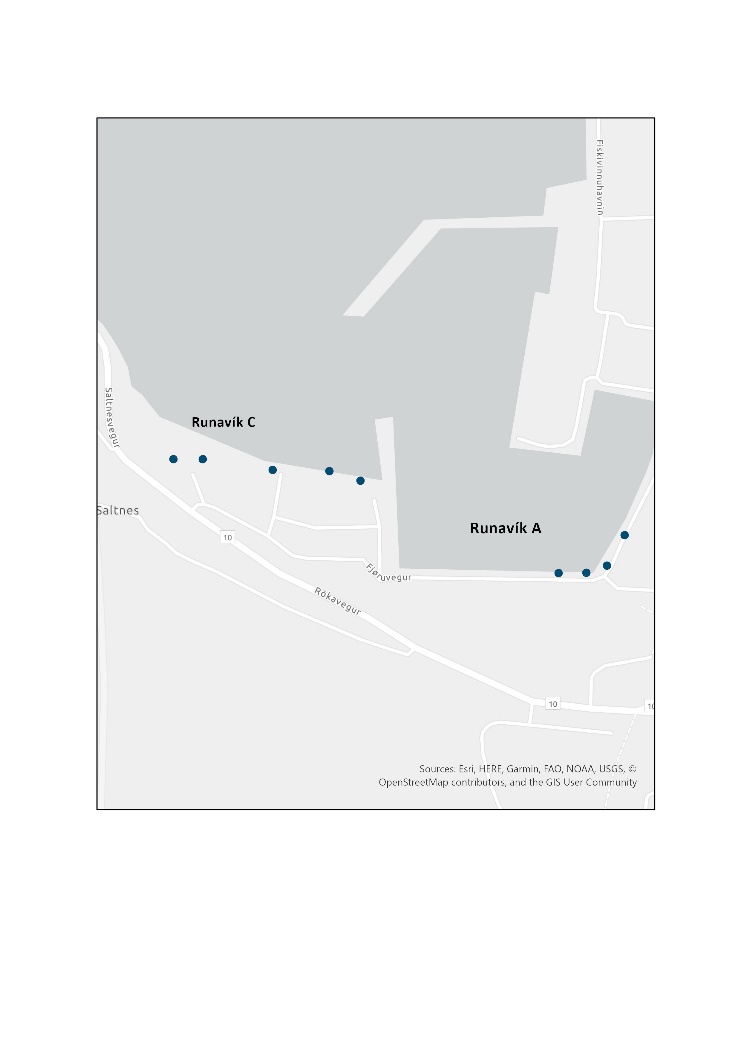 | 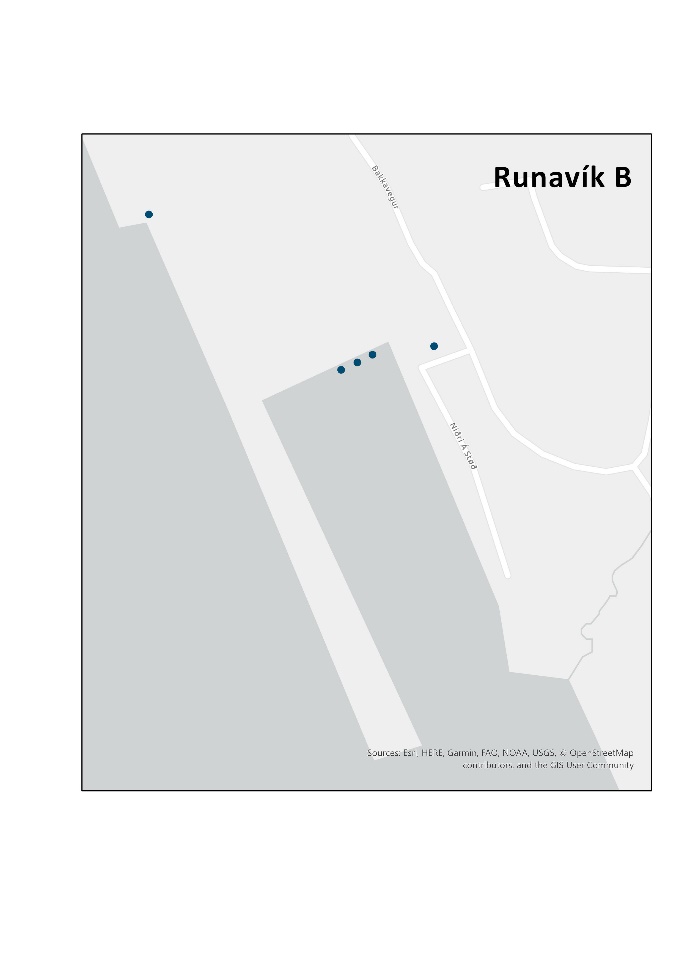 |
| 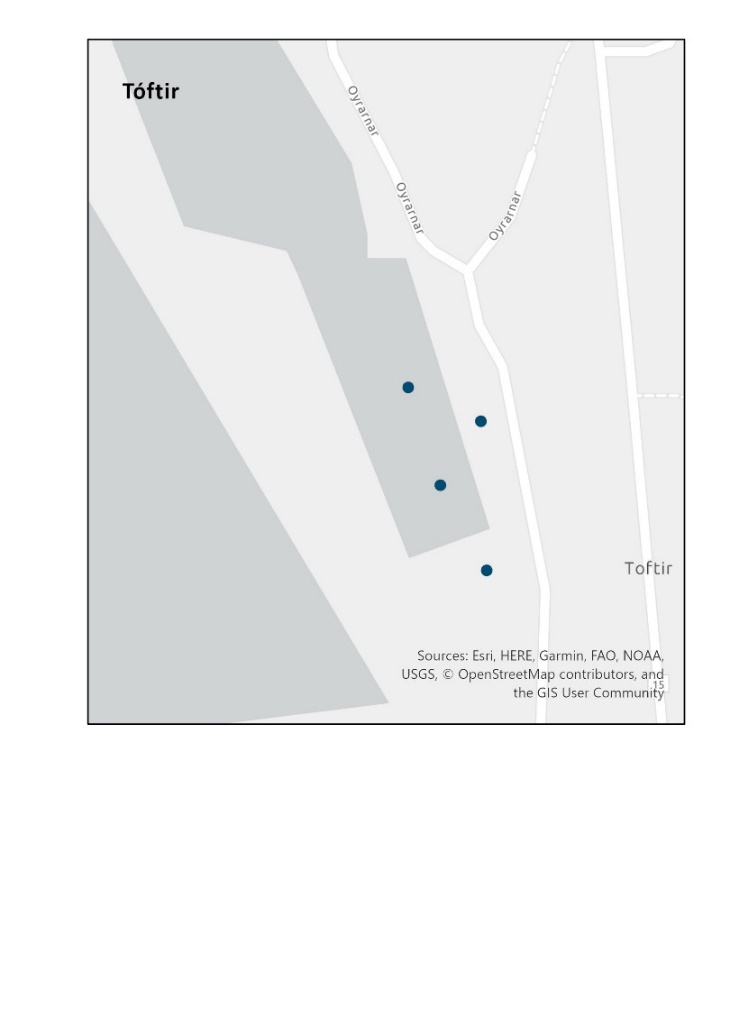 | 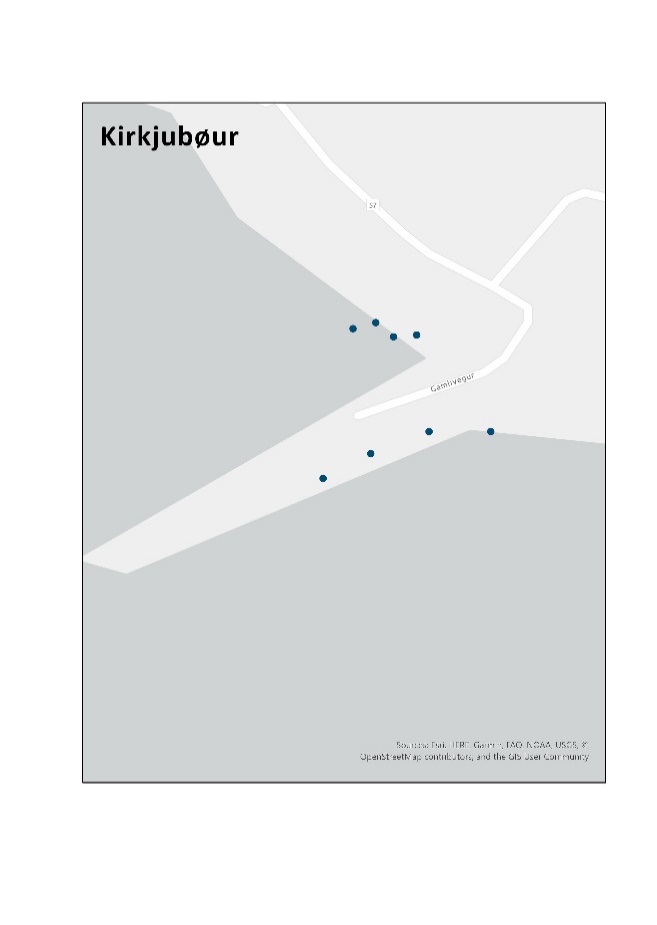 |

Figure S1.1: Sub samples taken at each sampling location

Table S1.1: Sampling locations coordinates, WGS84 projection

| Name | Latitude | Longitude |
| --- | --- | --- |
| Torshavn 1 | 62.00787 | -6.770214 |
| Torshavn 2 | 62.00841 | -6.773925 |
| Torshavn 3 | 62.00571 | -6.770985 |
| Torshavn 4 | 62.01038 | -6.756584 |
| Runavik 1 | 62.10979 | -6.724555 |
| Runavik 2 | 62.11048 | -6.729746 |
| Runavik 3 | 62.12540 | -6.727501 |
| Kirkjubøur | 61.95159 | -6.794128 |
| Toftir | 62.09516 | -6.739902 |

# S2. Species of seaweed

c)


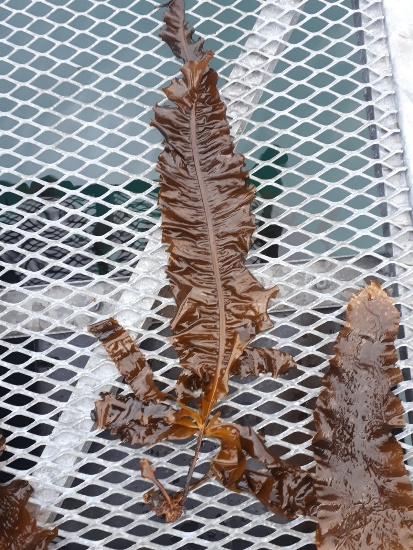


b)


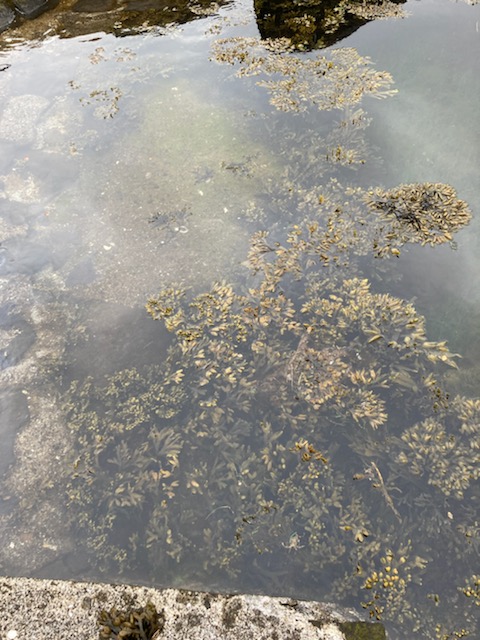


a)

*
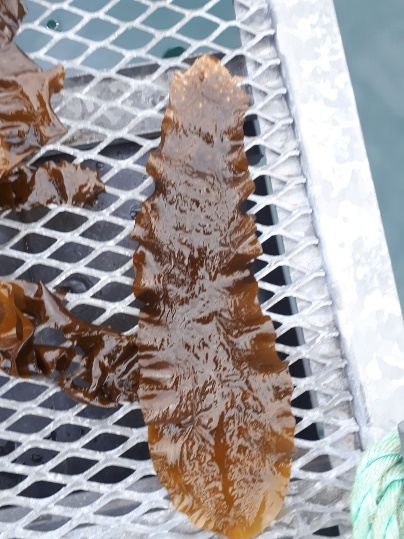
*

*Figure S2.1: seaweed sampled in this study: a) Saccharina latissima, b) Fucus c) Alaria Esculenta*

# S3. Method validation, QC and detection limit (DL)

##### S3.1 Recoveries of internal standard, precision and accuracy

The low recoveries of the internal standards (IS) in figure S3.1.1 did not lead to a systematic over or underestimation of the PAH concentrations. The accuracy was >90% for the PAHs >DL. This reassures the quality of the internal standard correction (figure 3.1.2). The inter-day precision reflected by the RSD values of the QC 1 is between 2% and 7% as shown in table S3.1.1.


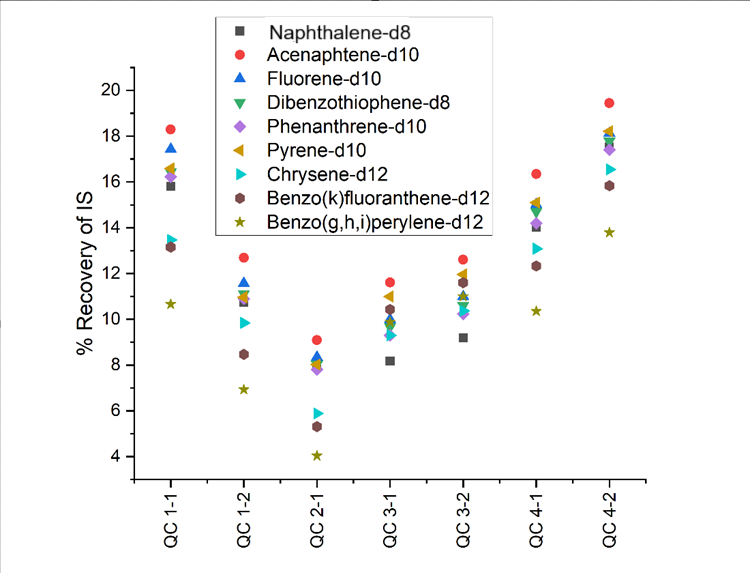


*Figure S3.1.1: Recovery of IS in QC1 samples.*

The recovery might be higher for the alkylated PAHs, as they are less polar, and thereby has a higher solubility in hexane compared to the non-alkylated homologues. Thus, the concentration of the alkylated PAHs may be slightly overestimated due to overcorrection by the non-alkylated PAH internal standards.


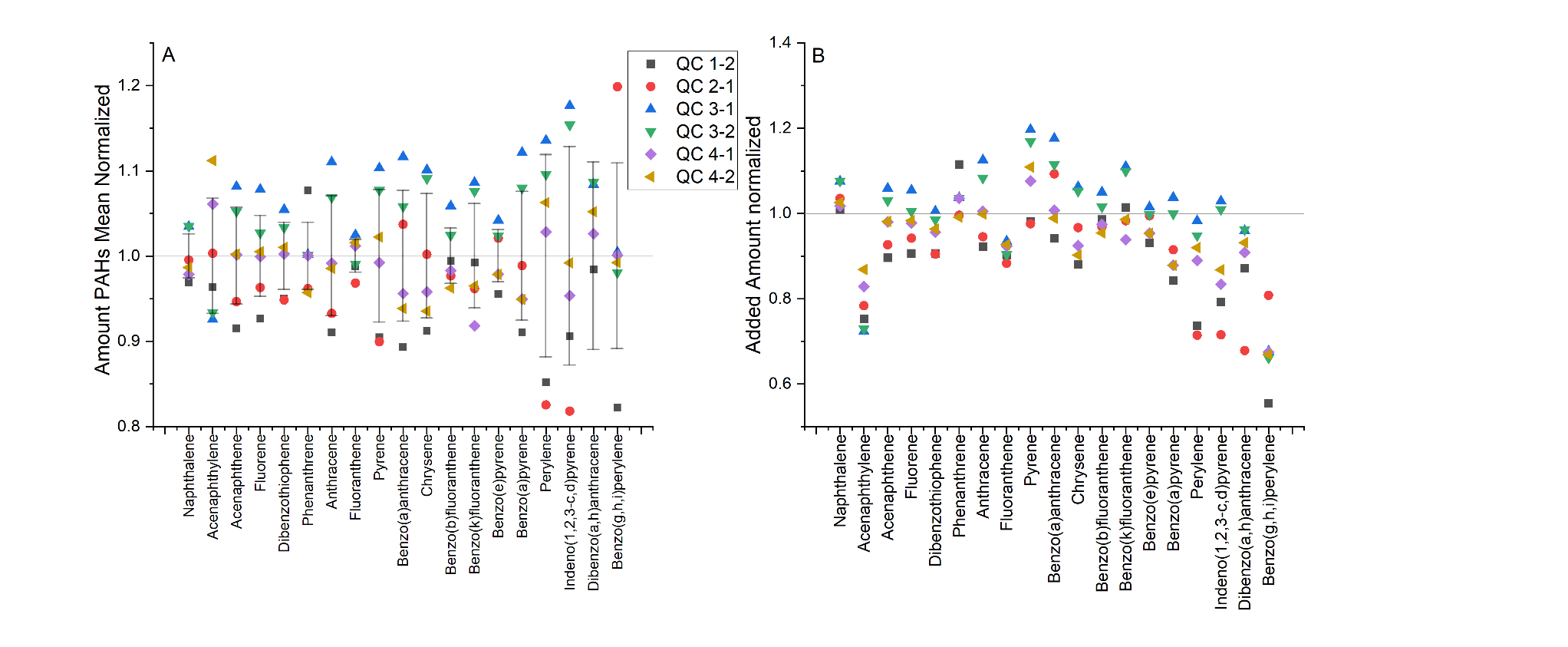
 *Figure S3.1.2: Recovery of spiked PAHs in QC1 samples corrected for losses using the corresponding internal standards.*

*Table S3.1.1: Accuracy, standard deviation (Stdev) and relativ standard deviation (rStdev) for selected PAHs measured in the spiked QC 1 samples*


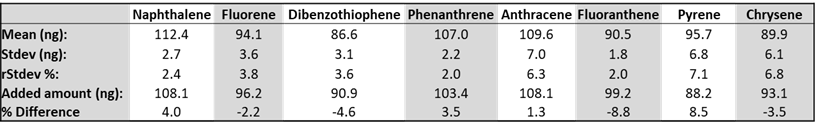


##### S3.2 Detection Limit (DL) and Limit of Quantification (LOQ)

The five samples of *Fucus* spec. used for intra-day precision were also used to calculate DLs and LOQs. The standard deviation for each of the PAHs in the five samples was multiplied with the average concentration of the respective PAH in the method blanks of batch 4 (eq. (1) and (2)).

DL = x̄[MBl] + s(QC) ∗ 3 equation 1

LOQ = x̄[MBl]+ s(QC) ∗ 10 equation 2

DL and LOQ values perform comparable or better than a method used for measuring PAHs in tree leaves which states an average accuracy of 86% and method detection limit (MDL) of between 0.1 to 4.9 ng/g dry weight (Jin et al., 2020). Since the DL is given per g WW in this study and with an approximated water content of 82% in the samples the MDL is most likely lower for this method.

# S4. Determination of water content - method

Dry weight was determined for selected samples. Two samples of each genus were weighted and dried in an oven at a 100°C for 20 hours and the weight was measured. The dry weight is not measured for all samples and therefore not used for weight correcting measured concentrations.

*Table S4.1: water and dry matter content in percentage*

|  | Mean water% | SD water | Mean dw% | SD dw% |
| --- | --- | --- | --- | --- |
| *Fucus* | 83 | 1.8 | 17 | 1.8 |

# S5. PAHs in standard solutions

*Table S5.1: Calibration standards*

| Calibration standards | |
| --- | --- |
| Parent compounds | **Alkylated compounds** |
| Naphthalene | 1-Methylnaphthalene |
| Dibenzothiophene | 2-Methylnaphthalene |
| Anthracene | 2,6-Dimethylnaphthalene |
| Phenanthrene | 2,3,5-Trimethylnaphthalene |
| Fluoranthene | 1,4,6,7-Tetramethylnaphthalene |
| Chrysene | 1-Methylfluorene |
| Pyrene | 1,7-dimethylfluorene |
| Benzo(a)anthracene | 2-Methylphenanthrene |
| Benzo(a)pyrene | 3,6-Dimethylphenanthrene |
| Perylene | 1,2,6-trimethylphenanthrene |
| Benzo(ghi)perylene | 1,2,6,9-Tetramethylphenanthrene |
| Benzo(k)fluoranthene | 4-Methylchrysene |
| Dibenz(a,h)anthracene | 6-Ethylchrysene |
| Fluorene | 1,3,6-Trimethylchrysene |
| Benzo(e)pyrene | 1-methylpyrene |
| Acenaphthene | 4,5-Dimethylpyrene |
| Acenaphthylene | 3-Methylbenzothiophene |
| Benzo(b)fluoranthene | 2-Methylthianaphthene/2-methyl-benzothiophene |
| Indeno(1,2,3-c,d)pyrene | 4-Methyldibenzothiophene |
|  | 4,6-Dimethyldibenzothiophene |
|  | 4-Ethyl-6-methyldibenzothiophene |

*Table S5.2: Internal and recovery standards*

| Internal standards | Recovery standards |
| --- | --- |
| Naphthalene-d8 | Acenaphthylene-d8 |
| Dibenzothiophene-d8 | Anthracene-d10 |
| Acenaphthene-d10 | Fluoranthene-d10 |
| Phenanthrene-d10 | Benz(a)anthracene-d12 |
| Pyrene-d10 | Benzo(a)pyrene-d12 |
| Fluorene-d10 | Indeno(1,2,3-c,d)pyrene-d12 |
| Chrysene-d12 |  |
| Benzo(k)fluoranthene-d12 |  |
| Benzo(g,h,i)perylene-d12 |  |

*Table S5.3: PAHs and their corresponding internal and recovery standard*

| PAHs | Internal standard | Recovery standard |
| --- | --- | --- |
| Naphthalene  C1-Naphthalene  C2-Naphthalene  C3-Naphthalene  C4-Naphthalene | Naphthalene-d8 | Acenaphthylene-d8 |
| Acenaphthylene | Acenaphthene-d10 |  |
| Fluorene | Flourene-d10 | Anhtracene d-10 |
| Dibenzothiophene | Dibenxothiophene-d10 |  |
| Anthracene  Phenanthrene  C1-Phenanthrene  C2-Phenanthrene  C3-Phenanthrene  C4-Phenanthrene | Phenanthrene-d10 |  |
| Pyrene  Fluoranthene | Pyrene-d10 | Fluoranthene-d10 |
| Chrysene | Chrysene-d12 | Benzo(a)anthracene-d12 |
| Benzo(b)fluoranthene | Benzo(k)fluoranthene-d12 |  |
| Benzo(bk)fluoranthene  Benzo(e)pyrene  Benzo(a)pyrene  Perylene |  |  |
| Dibenzo(a,h)anthracene | Benzo(g,h,i)perylene-d12 | Ideno(1,2,3-c,d)pyrene-d12 |
| Benzo(g,h,i)perylene |  |  |
| Ideno(1,2,3-c,d)pyrene |  |  |

*Table S5.4: mean concentration of analytes, internal standard and recovery standard in the quantitative standard*

|  | µg/mL | | | | | |
| --- | --- | --- | --- | --- | --- | --- |
|  | **Std1** | **Std2** | **Std3** | **Std4** | **Std5** | **Std6** |
| PAHs | 0.0039 | 0.012 | 0.024 | 0.048 | 0.079 | 0.16 |
| Alkylated PAHs | 0.0040 | 0.012 | 0.024 | 0.048 | 0.079 | 0.16 |
| Internal standards | 0.0081 | 0.024 | 0.049 | 0.081 | 0.16 | 0.24 |
| Acenaphthylene-d8 | 0.34 | 0.34 | 0.34 | 0.34 | 0.34 | 0.34 |
| Anthracene-d10 | 0.28 | 0.28 | 0.28 | 0.28 | 0.28 | 0.28 |
| Fluoranthene-d10 | 0,29 | 0,29 | 0,29 | 0,29 | 0,29 | 0,29 |
| Benz(a)anthracene-d12 | 0,31 | 0,31 | 0,31 | 0,31 | 0,31 | 0,31 |
| Benzo(a)pyrene-d12 | 0,30 | 0,30 | 0,30 | 0,30 | 0,30 | 0,30 |
| Indeno(1,2,3-c,d)pyrene-d12 | 0,30 | 0,30 | 0,30 | 0,30 | 0,30 | 0,30 |

Table S5.5: concentration of PAH_19_ in the standard solution

| **Compound** | **Concentration [mg/mL]** |
| --- | --- |
| Dibenzothiophene | 0,095 |
| Anthrancene | 0,113 |
| Phenanthrene | 0,108 |
| Fluoranthene | 0,103 |
| Chrysene | 0,097 |
| Pyrene | 0,092 |
| Benz(a)anthracene | 0,095 |
| Benzo(a)pyrene | 0,113 |
| Perylene | 0,093 |
| Benzo(g,h,i)perylene | 0,107 |
| Benzo(k)fluoranthene | 0,044 |
| Dibenz(a,h)anthracene | 0,095 |
| Fluorene | 0,100 |
| Benzo(e)pyrene | 0,088 |
| Acenaphthene | 0,110 |
| Acenaphthylene | 0,093 |
| Benzo(b)fluoranthene  Naphthalene | 0,097  0,113 |

# S6. Method development

For the method development *Fucus vesiculosus* sampled in Svanemøllen Harbor in Copenhagen was used. The homogenisation was carried out using a small kitchen blender (Køkkenchef minihakker, 500 mL, 300 W, item no. 10069036).

Extraction and clean-up was done based on the QuECHERS method (Anastassiades et al., 2003) but modified to fit analysis of PAHs in highly pigmented fruits and vegetables with a 20-80% water content (Słowik-Borowiec et al., 2022). To examine the most efficient extraction solvent acetonitrile and *n*-hexane:acetone 4:1 (v/v) were tested. Four samples were prepared; two with ACN and 2 with *n*-hexane:acetone 4:1 (v/v). One of each was placed in an ultrasonication bath, and blended with an Ultra Turrax (IKA, T 25 digital ULTRA-TURRAX®) to test the most efficient extraction method. The results showed that the ultrasonicated samples extracted with ACN had the highest PAH concentration.

The extracts had a bright green colour, due to co-extraction of pigments from the seaweed. To sufficiently clean up the sample from pigments without removing PAHs an experiment was conducted where three sorbents (C18 fused silica, Graphitised Carbon Black (GCB) and Florisil^Ⓡ^ were tested on extracts with both ACN and *n*-hexane:acetone 4:1. The remaining extracts from the previous experiment were spiked with 50 µl of a PAH stock solution, and an aliquot of 0.66 mL was transferred to a centrifuge tube, and added either 3 mg GCB, 60 mg Florisil^Ⓡ^ or 10 mg C-18. After mixing, the colour of the extract was examined to determine if pigment clean-up was sufficient. If the extract was not colourless the treatment was repeated in a total of three times. The samples were analysed on the GC-MS and the results were evaluated. C-18 was not successful at removing pigments, it only removed the PAHs. GCB was effective at removing pigments but in the process more than 80% of the four to five ring PAHs were also removed. Florisil^Ⓡ^ was efficient at removing pigments without affecting the PAH concentration, but mainly in the hexane:acetone 4:1 (v/v) extraction solvent. Based on this knowledge, it was concluded, that Florisil^Ⓡ^ was the most efficient sorbent for the removal of pigments.

**

*Figure S6.1: Visualisation of pigment clean-up. The graphs show how much of the initial pyrene and benzo(a)fluoranthene was lift in the extract after 1, 2 and, in case of GCB and C18, 3 times addition of sorbent.*


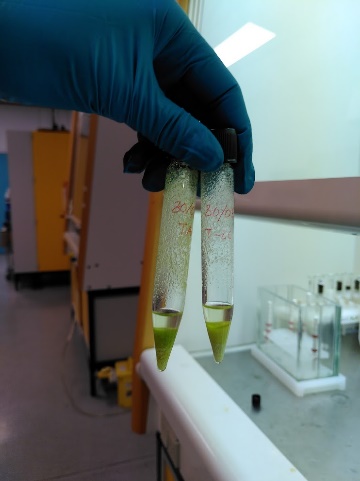


*Figure S6.2 After FlorisilⓇ clean- up*


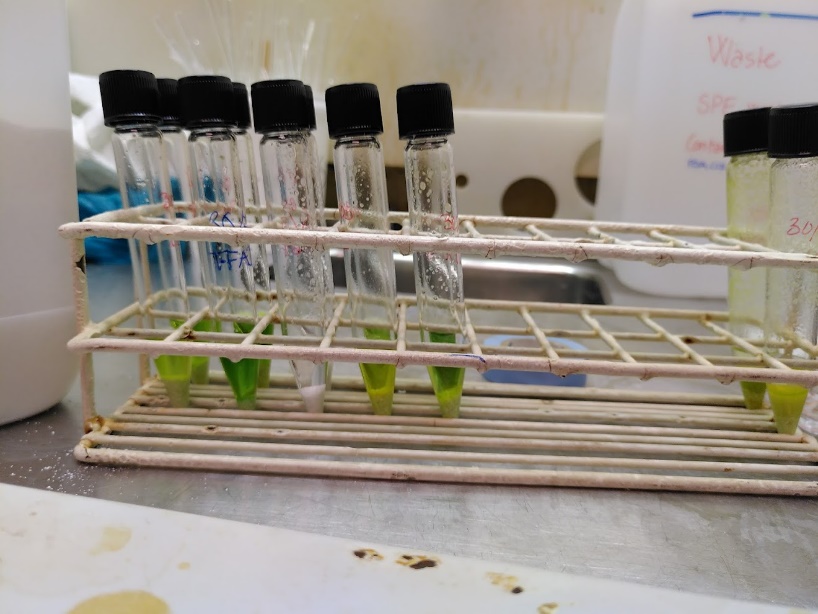


*Figure S6.1: Before FlorisilⓇ clean-up*

|  |  |
| --- | --- |

Samples were pre-concentrated to increase the sensitivity of the PAH analysis. One sample was extracted using 12.5ml *n*-hexane:acetone 4:1 (v/v). After extraction and clean-up the 12.5ml was evaporated to approx. 1mL at 40°C without nitrogen-flow and PAH were analysed in the extract using GC-MS. Another sample was extracted using 12.5ml acetonitrile, but before the clean-up was conducted, the ACN-fraction (approx. 10 mL) was mixed with 1 mL hexane and 10 mL milliQ water, and vortexed for 1 min. The *n*-hexane phase was transferred into a 15 mL centrifuge tube, cleaned up with Florisil^Ⓡ^ and PSA, and analysed on the GC-MS. The conclusion from the pre-concentration experiment was that the back extraction with n-hexane in ACN resulted in the most efficient pre-concentration. The theoretical up concentration factor of 12.5x based on the volume ratio was not observed when examining the amount of internal standards present in the final volume. The observed pre-concentration ranged from two to five times depending on the number of aromatic rings with lower pre-concentration of the PAHs with more aromatic rings. This should be accounted for in the quantification of the PAHs by the internal standard corrections.

Simultaneously an exhaustive extraction was performed, to see whether one extraction was sufficient for the extraction of PAHs. 12.5g of homogenized seaweed was mixed with 12.5 ml of either acetonitrile or hexane:acetone 4:1 (v/v) in 50 ml glass centrifuge tubes with 80 uL internal standard and ultrasonicated for 30 min. After sonication the supernatant was transferred to another 50 mL centrifuge tube, and the experiment was repeated for a total of three times. All extracts were analysed on the GC-MS, and the results showed that the largest fraction of PAHs was extracted in the 1^st^ extraction where the rest would be corrected for loss by the internal standard.

# S7 Chemical analysis

Table S7.1 Start times and m/z values for the SIM groups.

| SIM group | Start time (min) | m/z values |
| --- | --- | --- |
| 1 | 6.69 | 83, 85, 105, 123, 128, 134, 136, 138, 148, 152, 166, 180, 194 |
| 2 | 7.70 | 83, 85, 105, 123, 142, 148, 152, 154, 162, 166, 168, 180, 194 |
| 3 | 8.74 | 83, 85, 105, 123, 152, 154, 156, 160, 162, 164, 168, 180, 194 |
| 4 | 9.75 | 83, 85, 105, 123, 154, 164, 168, 170, 176, 182, 184, 190, 196 |
| 5 | 10.74 | 83, 85, 105, 123, 166, 170, 176, 180, 182, 184, 190, 192, 196 |
| 6 | 12.56 | 83, 85, 105, 123, 178, 182, 184, 188, 192, 194, 196, 198, 208 |
| 7 | 13.42 | 83, 85, 105, 192, 194, 196, 198, 202, 206, 208, 212, 220, 226 |
| 8 | 15.02 | 83, 85, 105, 202, 206, 212, 216, 217, 220, 226, 234, 240, 244 |
| 9 | 16.49 | 83, 85, 105, 191, 202, 206, 216, 217, 220, 226, 230, 234, 240 |
| 10 | 17.18 | 83, 85, 105, 191, 217, 218, 228, 230, 232, 234, 240, 242, 248 |
| 11 | 19.09 | 83, 85, 105, 191, 217, 218, 231, 242, 248, 252, 256, 264, 270 |
| 12 | 21.99 | 83, 85, 105, 191, 217, 218, 231, 252, 264, 270, 276, 278, 288 |

# S8. Relative standard deviation from paired samples

This table should be seen with the following points in mind. The standard deviations were calculated based on two samples. The samples taken at different timepoints also include the variance of being taken at different sub locations and the relative standard deviations are disproportionally influenced by noise for concentrations close to the detection limit.

In order to get a measure of the distribution of PAH concentrations the relative standard deviations were calculated for samples grouped under two categories: close proximity samples taken the same day and close proximity samples taken on different days (and different tide levels). To make an easy comparison the average relative standard deviation of all nonalkylated PAHs that were detected in those samples was calculated. The samples THA 1-4 from Thorshavn location A and RA1+2 from Runavik location A were grouped either with the sample taken at the same location and same timepoint (pairs THA1:THA2 and THA3:THA4) or grouped with a sample taken at the same location but at a different time point. (pairs THA1:THA3 THA1:THA4 THA2:THA3 and THA2:THA4 plus RA1:RA2)

*Table S8.1 relative standard deviation for paired samples collected at different sub-locations but at the same time; and for paired samples collected at different sub-locations at different time points.*

| Paired samples taken at different sub locations | Average relative standard deviation of non-alkylated PAHs |
| --- | --- |
| THA1:THA2 | 0.14 |
| THA3:THA4 | 1.2 |
| Paired samples taken at different sub-locations and different time points | Average relative standard deviation of non-alkylated PAHs |
| THA1:THA3 | 1.02 |
| THA1:THA4 | 0.60 |
| THA2:THA3 | 1.03 |
| THA2:THA4 | 0.73 |
| RA1:RA2 | 1.06 |
